# Readmissions and costs among younger and older adults for targeted conditions during the enactment of the hospital readmission reduction program

**DOI:** 10.1186/s12913-021-06399-z

**Published:** 2021-04-26

**Authors:** Chi-Hua Lu, Collin M. Clark, Ryan Tober, Meghan Allen, Walter Gibson, Edward M. Bednarczyk, Christopher J. Daly, David M. Jacobs

**Affiliations:** Department of Pharmacy Practice, School of Pharmacy and Pharmaceutical Sciences, University at Buffalo, 316 Pharmacy Building, Buffalo, NY USA

**Keywords:** Readmissions, NRD, Younger adults, HRRP, Targeted conditions, Costs

## Abstract

**Background:**

The Hospital Readmissions Reduction Program (HRRP) was introduced to reduce readmission rates among Medicare beneficiaries, however little is known about readmissions and costs for HRRP-targeted conditions in younger populations. The primary objective of this study was to examine readmission trends and costs for targeted conditions during policy implementation among younger and older adults in the U.S.

**Methods:**

We analyzed the Nationwide Readmission Database from January 2010 to September 2015 in younger (18–64 years) and older (≥65 years) patients with acute myocardial infarction (AMI), heart failure (HF), pneumonia, and acute exacerbations of chronic obstructive pulmonary disease (AECOPD). Pre- and post-HRRP periods were defined based on implementation of the policy for each condition. Readmission rates were evaluated using an interrupted time series with difference-in-difference analyses and hospital cost differences between early and late readmissions (≤30 vs. > 30 days) were evaluated using generalized linear models.

**Results:**

Overall, this study included 16,884,612 hospitalizations with 3,337,266 readmissions among all age groups and 5,977,177 hospitalizations with 1,104,940 readmissions in those aged 18–64 years. Readmission rates decreased in all conditions. In the HRRP announcement period, readmissions declined significantly for those aged 40–64 years for AMI (*p* < 0.0001) and HF (*p* = 0.003). Readmissions decreased significantly in the post-HRRP period for those aged 40–64 years at a slower rate for AMI (*p* = 0.003) and HF (*p* = 0.05). Readmission rates among younger patients (18–64 years) varied within all four targeted conditions in HRRP announcement and post-HRRP periods. Adjusted models showed a significantly higher readmission cost in those readmitted within 30 days among younger and older populations for AMI (*p* < 0.0001), HF (*p* < 0.0001), pneumonia (*p* < 0.0001), and AECOPD (*p* < 0.0001).

**Conclusion:**

Readmissions for targeted conditions decreased in the U.S. during the enactment of the HRRP policy and younger age groups (< 65 years) not targeted by the policy saw a mixed effect. Healthcare expenditures in younger and older populations were significantly higher for early readmissions with all targeted conditions. Further research is necessary evaluating total healthcare utilization including emergency department visits, observation units, and hospital readmissions in order to better understand the extent of the HRRP on U.S. healthcare.

**Supplementary Information:**

The online version contains supplementary material available at 10.1186/s12913-021-06399-z.

## Background

Reducing avoidable and costly re-hospitalizations within 30 days of discharge among Medicare beneficiaries in the United States has become a consensus from governments and researchers [1]. The Hospital Readmissions Reduction Program (HRRP) was initially introduced as part of the Patient Protection and Affordable Care Act (PPACA) in 2010 with the goal of creating financial incentives to reduce readmission rates and improve transitional care among Medicare beneficiaries [2]. In October 2012, hospitals began incurring financial penalties if 30-day, all-cause, risk-standardized readmission rates were higher than expected for Medicare beneficiaries with the following conditions: acute myocardial infarction (AMI), heart failure (HF), and pneumonia. In 2014, the HRRP was expanded to target additional conditions including acute exacerbations of chronic obstructive pulmonary disease (AECOPD) [2]. The penalties associated with the HRRP increased to 3% of all Medicare payments for fiscal year (FY) 2015, and > 2500 hospitals will face HRRP penalties for FY 2020 [3].

Current data show a relationship between the HRRP and a reduction in readmissions for the targeted conditions among Medicare beneficiaries [2–4]. Zuckerman et al. reported a decrease in hospital-level readmission rates for targeted HRRP conditions among elderly Medicare beneficiaries from 2007 to 2015 (21.5 to 17.8%) [2]. Less is known about readmission trends for the targeted conditions in younger, non-Medicare populations, in whom these conditions are increasingly common. Readmissions in younger populations covered by Medicaid or private insurance account for a high percentage of all readmissions (21 and 12%, respectively), with estimated total costs of over $1.5 billion [4–6]. Previous studies on readmissions among younger populations have not included AECOPD, a major condition targeted by the HRRP, and are limited to select states and years [4, 7, 8]. Broader knowledge of readmissions and costs for the four major targeted conditions among a nationally representative cohort of younger patients may help tailor clinical initiatives and policy development.

Initiatives are being developed across the health continuum to improve readmission rates through the implementation of transition of care and readmission reduction programs [9–12]. These programs may include younger populations that are not included as part of HRRP policy. Understanding readmission trends and costs for younger and older populations from large database in the context of the HRRP-targeted conditions may incentivize improvements in these transition programs. Therefore, the objectives of this study were to: (i) examine readmission trends for HRRP-targeted conditions including AMI, HF, pneumonia, and AECOPD prior to and after implementation of the policy among younger and older populations in the United States and (ii) evaluate the hospital cost differences between early and late readmission events. This study is novel in the following ways: (i) readmission trends for HRRP targeted conditions were evaluated and stratified based on three different age groups (18–39, 40–64, ≥65 years of age) and (ii) we examined cost differences across the four conditions between early (≤30 days) and late (> 30 days) readmissions within younger and older populations.

## Methods

### Data source

We analyzed data from the Nationwide Readmission Database (NRD) developed by the Agency for Healthcare Research and Quality (AHRQ) for the Healthcare Cost and Utilization Project (HCUP) as it is a large-scale administrative database designed specifically to support analyses of national readmissions for all ages and payers [13]. The NRD contains year-specific verified patient linkage numbers that can be used to track persons across hospitals in a state within a calendar year. The database includes all discharge data from community hospitals in 27 states but not from rehabilitation or long-term acute care hospitals. The NRD is designed to generate national estimates of readmissions since it includes over half of the U.S. population and hospitalizations each year. For example, in 2015, the database included 17.2 million raw discharges and 36 million weighted discharges representing 56.6% of all U.S. hospitalizations. The University at Buffalo Institutional Review Board exempted this study from review, as data were de-identified and publicly available through the AHRQ.

### Study population

We included all adults ≥18 years with an *International Classification of Diseases, Ninth Revision, Clinical Modification* (ICD-9-CM) code for AMI, HF, and pneumonia between January 2010 and September 2015. For AECOPD, we only included adults aged ≥40 years, as this threshold has been used to help exclude patients with asthma exacerbations [14]. We also identified non-targeted conditions, representing hospitalizations excluding AMI, HF, pneumonia, and AECOPD as our control cohort. The ICD-9-CM codes were chosen based on those published by the Centers for Medicare and Medicaid Services (CMS) for the HRRP for assessment of all-cause readmissions (S1, S2, S3–4 Tables) [15–18]. We excluded patients if they died during the index hospitalization, discharged against medical advice, or if they were residents of a different state. As recommended by the HCUP, we used data for 11 months (January to November) to capture all 30-day follow-up for all patients from 2010 to 2014. We used data for nine months (January to September) in 2015 since *International Classification of Diseases, Tenth Revision, Clinical Modification* (ICD-10-CM) codes were implemented on October 1. We included subjects up to September 2015 given the substantial differences between ICD-9-CM to ICD-10-CM codes and lack of comparison of the two coding systems in the U.S.

### Patient and hospital covariates

The NRD classifies covariates as demographic, pre-existing comorbidity, clinical, and hospital characteristics. Patient demographic variables included were age, gender, insurance status, and median household income. Age was stratified into 18–39, 40–64, and ≥ 65 years groups for AMI, HF, pneumonia, and non-targeted conditions. For AECOPD, age was stratified into 40–64 and ≥ 65 years groups. Insurance status collected by the database was designated as the primary insurance payer for each admission. Insurance was categorized as Medicare, Medicaid, private insurance, self-pay, or other (including no charge). Income levels were based on the estimated median household income of residents in the patient’s zip code and were divided into $1–$41,999, $42,000–$51,999, $52,000–$67,999, and > $68,000. Pre-existing comorbidities were assessed as overall comorbidity burden using the Elixhauser comorbidities provided by the AHRQ [19]. Discharge disposition was categorized as home, home healthcare, or skilled nursing facility and other (including transfers to immediate care, another type of facility, or discharged alive but destination unknown). Hospitalization characteristics included length of stay in days and hospital charges associated with the index hospitalization and readmission.

### Outcomes

The primary outcome of this study was 30-day all-cause unplanned readmission rate. We followed the CMS methodologies for condition specific readmission measures to construct our analytical cohorts (AMI, HF, pneumonia and AECOPD) for each age group [15–18]. Briefly, the CMS measures only define the first readmission within 30 days of discharge as a 30-day readmission. Additional readmissions within the 30-day period are not counted as 30-day readmissions or index hospitalizations for the same condition. After 30 days from discharge, hospitalizations are counted as index admissions if they meet the inclusion criteria. The secondary outcome was costs of index hospitalization and readmission. Costs were defined as the amount that hospitals billed for services and were standardized to 2015 dollars using the index from the U.S. Bureau of Labor Statistics [20]. We evaluated differences in readmissions costs by stratifying readmissions as either an early (≤30 days) or late readmission (> 30 days) and further stratified by age groups including 18–39, 40–64, and ≥ 65 years. Late readmissions were defined patients re-entered to hospitals above 30 days up to the end of each year due to the nature of NRD.

### Statistical analysis

To account for the complex survey design of the NRD, we used a survey-specific methodology with hospital as clusters, NRD stratum as strata, and discharge-level weights as weights to obtain study population data and weighted nationwide overall and annual 30-day readmission rates for each targeted condition. Demographic and clinical characteristics were compared between the targeted conditions. Categorical variables were reported as percentages and compared using the chi-square test and continuous variables were reported as medians (interquartile ranges, IQR) and compared using the Mann-Whitney U test.

We first evaluated overall changes including all ages and then stratified the data into three age-specific groups (18–39, 40–64, ≥65 years). Readmission rates were evaluated using a segmented regression analysis of interrupted time series (ITS) data to assess changes in level and slope of the regression lines in the HRRP announcement phase and post-HRRP segments. This analysis allowed the estimation of changes in outcomes between HRRP announcement and post-HRRP phases while accounting for both sudden changes and changes in trends for the outcome of interest. A change in level would indicate an immediate effect of the HRRP on the outcome of interest, while a change in slope (or trend) of the regression line would point to a longer, more sustained response for the outcome measured. Then, we expanded our segmented regression models with the difference-in-difference (DD) approach among younger and older populations separately [21, 22]. We calculated the difference in rates (e.g. the rate of AMI in 18–39 age group minus the rate of non-targeted conditions in 18–39 age group for every time period) for each targeted condition [21]. Taking the difference allowed us to collapse the 2 time series into 1 in order to estimate a DD effect and also estimate how the change in readmissions rates of each targeted condition differed from non-targeted conditions over the same time period.

The ITS analysis is a quasi-experimental approach for evaluating longitudinal effects of policy interventions, and the interventions are different for each condition [23, 24]. For AMI, HF, and pneumonia, the HRRP announcement phase was defined from January 1, 2010 to September 30, 2012 and the post-HRRP phase included October 1, 2012 to August 31, 2015. For AECOPD, the HRRP announcement phase included January 1, 2010 to September 30, 2014 and the post-HRRP phase included October 1, 2014 to August 31, 2015. We examined monthly outcome measures for the entire study peroid for each condition and this included 63 observation points. Overall, the equation is represented as follows:

$$ {Y}_t={\beta}_0+{\beta}_1\ast {time}_t+{\beta}_2\ast HRRP\ implementation+{\beta}_3\ast time\ after\ {HRRP\ implementation}_t+{e}_t $$

*Y*_*t*_ is our outcome measure, the rate of readmissions per month in month *t*; *time*_*t*_ is a continous variable (1–63 months), indicating time in month *t* starting from January 2010 to August 2015. *HRRP implementation* is a dummy indicator for pre or post HRRP implmentation (before = 0, after = 1); and the *time after HRRP implementation*_*t*_ is a continous variable counting the number of months after the HRRP implementation at time *t* (before = 0). *β*_0_ estimates the baseline level of the outcome (such as the rate of readmissions per month); *β*_1_ estimates the change in the rate of readmissions per month occuring in each month before HRRP implementation; *β*_2_ estimates the level change in the rate of readmissions per month immediately after HRRP implementation at the end of the preceding segment; and *β*_3_ estimates the change in the trend in the rates of readmissions per month after the HRRP implementation, compared to the trend before the implementation. The error term e_t_ includes random error and autocorrelation. All models were adjusted for seasonality. The sum of *β*_1_ and *β*_3_ was calculated to estimate the post-HRRP implementation slope, and the Wald test was used for hypothesis testing [24].

Index hospitalization and readmission costs for each patient were calculated using standard methods described by the HCUP. These costs were adjusted for inflation to 2015 dollars using the medical component of the consumer price index, and all costs are reported in 2015 dollars [20]. We evaluated differences in readmission costs between early (≤30 days) and late (> 30 days) readmissions within each targeted condition using a generalized linear model with a log link function and gamma distribution. The cost model was then applied to each targeted condition and furhter stratified by previously defined age groups. Analyses were performed using SAS version 9.4 (SAS Institute, Cary, NC), and all hypothesis testing was two sided with a significance set at *p* < 0.05.

## Results

Between January 2010 and September 2015, there were an estimated 2,384,531 hospitalizations nationally for AMI, 4,491,180 for HF, 5,810,738 for pneumonia, 4,198,163 for AECOPD, and 93,742,090 for non-targeted conditions. There were an estimated 355,025 (14.89%) readmissions nationally for AMI, 1,079,016 (24.03%) readmissions for HF, 1,009,849 (17.38%) readmissions for pneumonia, 893,376 (21.28%) readmissions for AECOPD, and 12,564,709 (13.40%) for non-targeted conditions. The selected demographic characteristics are listed in Table 1. Patients with Medicare were the largest group, and most patients were discharged to home across the targeted and non-targeted conditions. Further breakdowns for patient characteristics of index admissions by age, insurance, and condition are listed in S5 Table.

**Table 1:** Demographic and Clinical Characteristics of Index Admissions among Targeted and Non-targeted Condition within the Nationwide Readmissions Database, 2010–2015

Characteristics, No. (%)	AMI	HF	Pneumonia	COPD	Non-targeted Conditions
**Age, year, median (IQR)**	67 (57, 78)	75 (63, 84)	73 (59, 83)	68 (59, 77)	58 (38, 74)
18–39	56,036 (2.3)	104,915 (2.3)	379,382 (6.5)	–	24,332,212 (26.0)
40–64	1,009,250 (42.3)	1,155,234 (25.7)	1,620,305 (27.9)	1,652,056 (39.4)	32,378,953 (34.5)
≥ 65	1,319,245 (55.4)	3,231,031 (72.0)	3,811,051 (65.6)	2,546,107 (60.6)	37,030,924 (39.5)
**Gender**					
Male	1,469,081 (61.6)	2,250,158 (50.1)	2,813,803 (48.4)	1,749,810 (41.7)	38,694,908 (41.3)
Female	915,450 (38.4)	2,241,023 (49.9)	2,996,935 (51.6)	2,448,353 (58.3)	55,047,181 (58.7)
**Insurance**					
Medicare	1,358,056 (57.1)	3,416,220 (76.1)	4148,871 (71.5)	2,961,680 (70.7)	43,019,710 (46.0)
Medicaid	165,552 (7.0)	392,824 (8.8)	514,536 (8.9)	515,798 (12.3)	16,644,864 (17.8)
Private	618,579 (26.0)	444,947 (10.0)	816,310 (14.1)	461,905 (11.0)	24,383,243 (26.0)
Self-pay	141,036 (5.9)	125,322 (2.8)	175,595 (3.0)	129,210 (3.1)	93,642 (5.8)
Other	95,782 (4.0)	102,929 (2.3)	144,875 (2.5)	121,275 (2.9)	78,877 (4.4)
**Median household income**					
$1–$41,999	722,329 (30.8)	1,521,192 (34.4)	1,803,527 (31.5)	1,542,186 (37.3)	28,990,950 (31.4)
$42,000–$51,999	625,069 (26.7)	1,120,857 (25.3)	1,486,496 (26.0)	1,115,493 (27.0)	23,155,075 (25.1)
$52,000–$67,999	551,102 (23.5)	985,561 (22.3)	1,323,968 (23.1)	874,642 (21.2)	21,546,830 (23.3)
≥ $68,000	446,519 (19.0)	798,166 (18.0)	1,105,383 (19.4)	600,507 (14.5)	18,598,519 (20.2)
**Discharge location**					
Home	1,703,678 (71.5)	2,407,431 (53.6)	3,044,937 (52.4)	2,743,387 (65.3)	66,911,595 (71.4)
Home healthcare	310,865 (13.0)	1,137,616 (25.3)	1,039,137 (17.9)	815,016 (19.4)	11,284,045 (12.0)
SNF/other	369,988 (15.5)	946,133 (21.1)	1,726,664 (29.7)	639,760 (15.3)	15,546,448 (16.6)
**No. of comorbidities, median (IQR)**	7 (5, 9)	8 (6, 10)	6 (4,8)	7 (5, 9)	4 (2,7)
**Length of stay in days, median (IQR)**	3 (2, 5)	4 (2,6)	4 (3,7)	4 (2,6)	3 (2,5)
**Total cost for index admission, median (IQR)**	$57,610 ($33,195, $95,207)	$25,024 ($14,514, $45,047)	$26,433 ($15,068, $48,317)	$22,512 ($13,382, $39,122)	$22,230 ($12,415, $41,723)

*Abbrev*. *AMI* acute myocardial infarction, *HF* heart failure, *COPD* chronic obstructive pulmonary disease, *IQR* interquartile range

### Readmission trends among targeted conditions prior to and after HRRP implementation

#### Acute myocardial infarction

##### Older population (≥ 65 years)

**Fig. 1:**
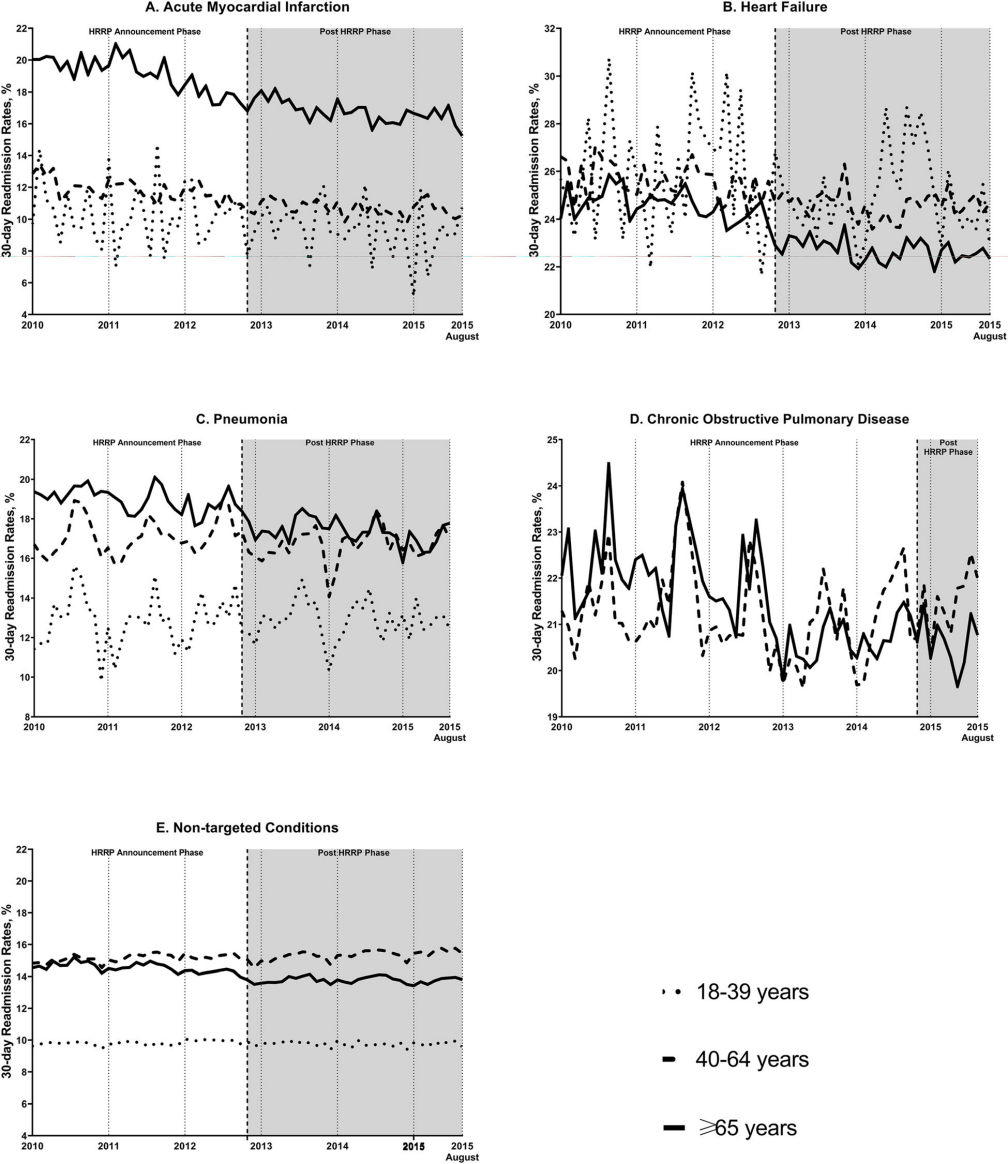
Trends in 30-day Readmission Rates Stratified by Age Groups and Targeted/Non-targeted Condition

**Fig. 2:**
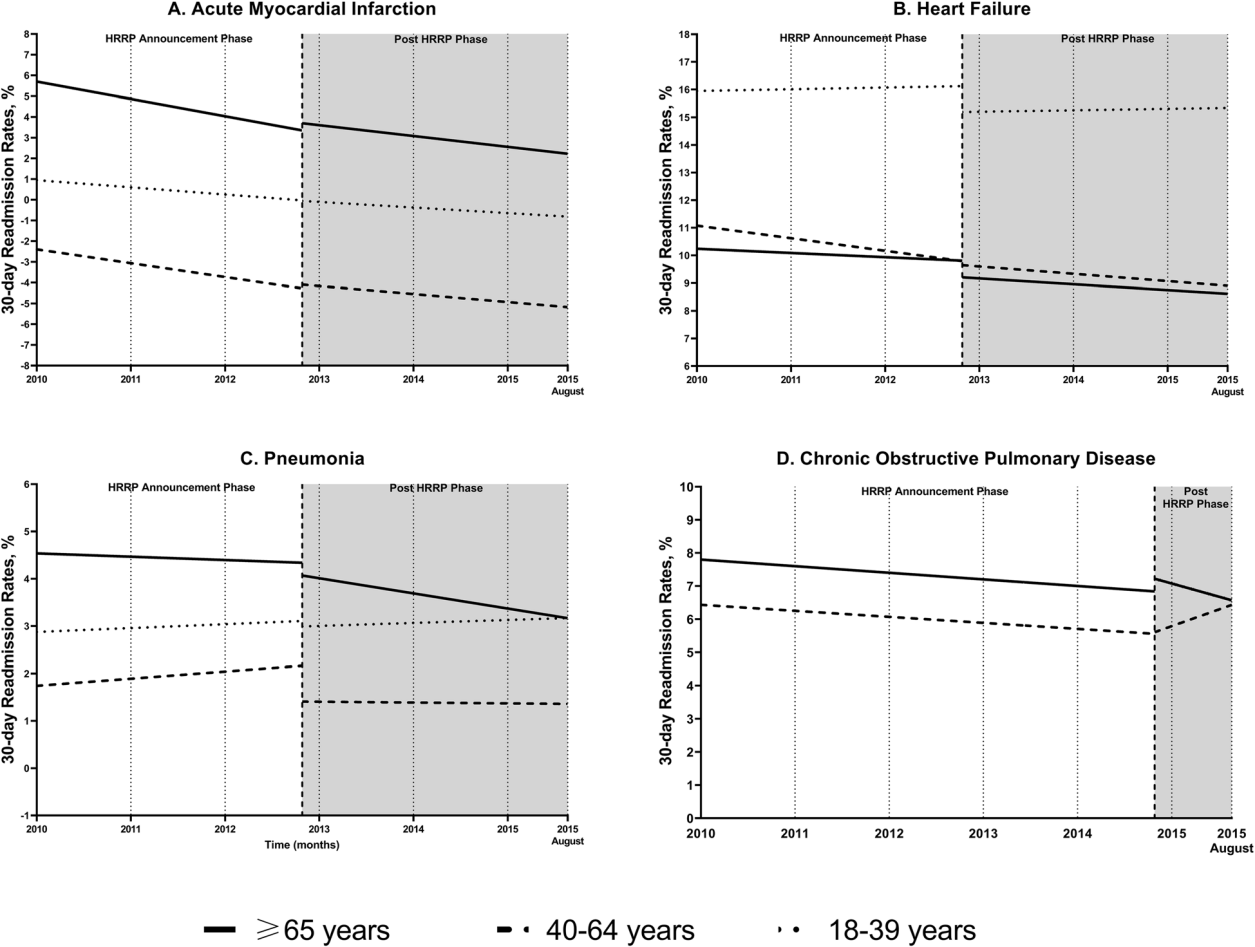
DD Estimate of Change in 30-day Readmission Trends Prior to and After the Implementation of the Hospital Readmissions Reduction Program Stratified by Targeted Condition and Age Groups using Interrupted Time Series Analyses

The 30-day readmission rates among aged ≥65 years decreased from 19.79% in 2010 to 16.56% in 2015 for AMI (Table 2**,** Fig. 1**a**). The slope decreased in the HRRP announcement (slope: − 0.0915, *p* < 0.0001) and post-HRRP (slope: − 0.0433, p < 0.0001) phases (Table 3). The slope in the HRRP announcement phase was slightly greater compared to the slope in the post-HRRP phase, indicating readmission rates decreased at a slower rate during the post-HRRP phase (difference: 0.0482, *p* = 0.006). From our DD models, readmission rates declined significantly in the HRRP announcement phase (slope: − 0.0676, *p* < 0.0001), and the rates continued to decrease after HRRP implementation (slope: − 0.0454, *p* = 0.001) (Table 4**,** Fig. 2**a**).

**Table 2:** Number of Index Admissions and 30-day Readmissions Stratified by Targeted and Non-targeted Condition and Age from the Nationwide Readmissions Database, 2010 to 2015^a^

Characteristics		2010	2011	2012	2013	2014	2015	Overall
**Acute Myocardial Infarction**								
All age groups	Total index admissions	401,418	400,230	416,330	422,930	418,275	325,349	2,384,531
	No. of 30-day readmissions	65,559	63,995	61,912	60,829	57,802	44,927	355,025
	**Readmission rates (%)**	**16.33**	**15.99**	**14.87**	**14.38**	**13.82**	**13.81**	**14.89**
18–39 years	Total index admissions	9938	9939	9466	9702	9392	7599	56,036
	No. of 30-day readmissions	1029	1017	963	965	874	663	5510
	**Readmission rates (%)**	**10.35**	**10.23**	**10.17**	**9.94**	**9.31**	**8.72**	**9.83**
40–64 years	Total index admissions	170,426	170,271	175,812	178,108	177,147	137,486	1,009,250
	No. of 30-day readmissions	20,773	20,129	19,886	19,541	18,562	14,711	113,602
	**Readmission rates (%)**	**12.19**	**11.82**	**11.31**	**10.97**	**10.48**	**10.70**	**11.26**
≥ 65 years	Total index admissions	221,054	220,020	231,052	235,120	231,737	180,263	1,319,246
	No. of 30-day readmissions	43,756	42,850	41,064	40,324	38,367	29,553	235,914
	**Readmission rates (%)**	**19.79**	**19.48**	**17.77**	**17.15**	**16.56**	**16.39**	**17.88**
**Heart Failure**								
All age groups	Total index admissions	785,712	767,318	751,847	767,218	790,018	629,067	4,491,180
	No. of 30-day readmissions	197,339	191,576	182,318	179,705	182,500	145,579	1,079,016
	**Readmission rates (%)**	**25.12**	**24.97**	**24.25**	**23.42**	**23.10**	**23.14**	**24.03**
18–39 years	Total index admissions	18,099	17,510	17,433	17,841	18,857	15,176	104,916
	No. of 30-day readmissions	4667	4507	4510	4315	4960	3658	26,617
	**Readmission rates (%)**	**25.79**	**25.74**	**25.87**	**24.19**	**26.30**	**24.10**	**25.37**
40–64 years	Total index admissions	198,110	194,402	192,074	197,902	206,895	165,851	1,155,234
	No. of 30-day readmissions	51,284	49,840	48,277	48,973	50,389	40,869	289,632
	**Readmission rates (%)**	**25.89**	**25.64**	**25.13**	**24.75**	**24.35**	**24.64**	**25.07**
≥ 65 years	Total index admissions	569,503	555,406	542,340	551,476	564,266	448,041	3,231,032
	No. of 30-day readmissions	141,389	137,229	129,530	126,417	127,150	101,052	762,767
	**Readmission rates (%)**	**24.83**	**24.71**	**23.88**	**22.92**	**22.53**	**22.55**	**23.61**
**Pneumonia**								
All age groups	Total index admissions	971,287	1,013,665	991,446	1,026,460	998,629	809,252	5,810,738
	No. of 30-day readmissions	176,961	181,312	175,709	174,425	167,920	133,522	1,009,849
	**Readmission rates (%)**	**18.22**	**17.89**	**17.72**	**16.99**	**16.82**	**16.50**	**17.38**
18–39 years	Total index admissions	64,872	67,535	66,124	64,078	67,980	48,793	379,382
	No. of 30-day readmissions	8248	8409	8489	8360	8461	6272	48,239
	**Readmission rates (%)**	**12.71**	**12.45**	**12.84**	**13.05**	**12.45**	**12.85**	**12.72**
40–64 years	Total index admissions	271,022	284,604	274,966	282,430	289,602	217,680	1,620,304
	No. of 30-day readmissions	45,963	47,826	47,133	46,934	47,716	36,277	271,849
	**Readmission rates (%)**	**16.96**	**16.80**	**17.14**	**16.62**	**16.48**	**16.67**	**16.78**
≥ 65 years	Total index admissions	635,392	661,525	650,355	679,952	641,047	542,779	3,811,050
	No. of 30-day readmissions	122,750	125,077	120,086	119,132	111,744	90,974	689,763
	**Readmission rates (%)**	**19.32**	**18.91**	**18.46**	**17.52**	**17.43**	**16.76**	**18.10**
**Chronic Obstructive Pulmonary Disease**								
All age groups	Total index admissions	729,546	757,553	730,901	733,946	697,041	549,175	4,198,163
	No. of 30-day readmissions	159,245	166,954	156,242	150,882	145,595	114,457	893,376
	Readmission rates (%)	**21.83**	**22.04**	**21.38**	**20.56**	**20.89**	**20.84**	21.28
40–64 years	Total index admissions	283,859	296,422	291,524	286,029	282,095	212,126	1,652,055
	No. of 30-day readmissions	60,293	64,070	61,161	59,086	59,441	45,440	349,491
	**Readmission rates (%)**	**21.24**	**21.61**	**20.98**	**20.66**	**21.07**	**21.42**	**21.15**
≥ 65 years	Total index admissions	445,687	461,131	439,377	447,918	414,946	337,049	2,546,108
	No. of 30-day readmissions	98,952	102,884	95,081	91,797	86,155	69,017	543,886
	**Readmission rates (%)**	**22.20**	**22.31**	**21.64**	**20.49**	**20.76**	**20.48**	**21.36**
**Non-targeted Conditions**								
All age groups	Total index admissions	16,591,979	16,575,452	16,602,556	16,186,040	16,021,104	11,764,959	93,742,090
	No. of 30-day readmissions	2,247,016	2,252,019	2,228,922	2,142,287	2,128,186	1,566,279	12,564,709
	**Readmission rates (%)**	**13.54**	**13.59**	**13.43**	**13.24**	**13.28**	**13.31**	**13.40**
18–39 years	Total index admissions	4,264,477	4,255,748	4,286,288	4,214,362	4,241,154	3,070,183	24,332,212
	No. of 30-day readmissions	416,414	415,862	425,751	412,567	412,243	300,072	2,382,909
	**Readmission rates (%)**	**9.76**	**9.77**	**9.93**	**9.79**	**9.72**	**9.77**	**9.79**
40–64 years	Total index admissions	5,796,067	5,782,105	5,770,024	5,561,777	5481,932	3,987,048	32,378,953
	No. of 30-day readmissions	869,102	881,487	875,343	846,110	844,815	619,742	4,936,599
	**Readmission rates (%)**	**14.99**	**15.25**	**15.17**	**15.21**	**15.41**	**15.54**	**15.25**
≥ 65 years	Total index admissions	6,531,435	6,537,600	6,546,243	6,409,902	6,298,017	4,707,728	37,030,925
	No. of 30-day readmissions	961,499	954,670	927,827	883,609	871,127	646,464	5,245,196
	**Readmission rates (%)**	**14.72**	**14.60**	**14.17**	**13.79**	**13.83**	**13.73**	**14.16**

^a^ Data are included up to August 31, 2015 due to initiation of ICD-10-CM codes in October 1, 2015 within the U.S. coding system

**Table 3:** Interrupted Time Series Analysis Evaluating the Change in 30-day Readmission Rates Prior to and After Implementation of the Hospital Readmission Reduction Program Stratified by Targeted/Non-targeted Condition and Age Group

	HRRP Announcement Phase Trend^**a**^ (SE)	***P***	Level Change^**b**^ (SE)	***P***	Post-HRR Phase Trend^**c**^	***P***	Difference in Trends^**d**^ (SE)	***P***
**Acute Myocardial Infarction**								
All age groups	−0.0697 (0.008)	**<.0001**	−0.2564 (0.198)	0.20	−0.0341	**<.0001**	0.0356 (0.01)	**< 0.001**
18–39 years	−0.0157 (0.026)	0.54	−0.2452 (0.643)	0.70	−0.0337	0.17	−0.018 (0.035)	0.61
40–64 years	−0.0385 (0.005)	**<.0001**	−0.2882(0.135)	**0.04**	−0.0159	**0.001**	0.0226 (0.007)	**0.001**
≥ 65 years	− 0.0915 (0.01)	**<.0001**	− 0.3971 (0.268)	0.14	− 0.0433	**<.0001**	0.0482 (0.013)	**0.006**
**Heart Failure**								
All age groups	− 0.0266 (0.009)	**0.004**	−1.0354 (0.225)	**<.0001**	−0.0126	0.14	0.014 (0.124)	0.26
18–39 years	0.0157 (0.037)	0.67	−1.3105 (0.961)	0.18	0.0196	0.58	0.0039 (0.048)	0.94
40–64 years	− 0.0209 (0.011)	0.07	− 0.6897 (0.293)	**0.02**	− 0.0024	0.82	0.0185 (0.015)	**0.022**
≥ 65 years	− 0.0275 (0.010)	**0.008**	−1.2214 (0.254)	**<.0001**	−0.017	0.06	0.0105 (0.014)	0.46
**Pneumonia**								
All age groups	−0.0186 (0.006)	**0.003**	−0.5478 (0.14)	**0.0003**	−0.0196	**< 0.001**	−0.001 (0.009)	0.91
18–39 years	0.0011 (0.015)	0.94	0.1852 (0.383)	0.63	−0.0077	0.59	−0.0088 (0.019)	0.65
40–64 years	0.0104 (0.009)	0.26	−0.6752 (0.236)	**0.006**	0.0111	0.19	0.0008 (0.011)	0.95
≥ 65 years	−0.0339 (0.007)	**<.0001**	−0.5158 (0.171)	**0.004**	−0.0328	**<.0001**	0.0011 (0.008)	0.89
**Chronic Obstructive Pulmonary Disease**								
All age groups	−0.0231 (0.013)	0.09	0.2827 (0.606)	0.64	−0.026	0.79	−0.0029 (0.095)	0.98
40–64 years	−0.0056 (0.015)	0.71	0.2427 (0.735)	0.74	0.0914	0.79	0.037 (0.115)	0.75
≥ 65 years	−0.0360 (0.012)	**0.005**	0.2882 (0.671)	0.67	−0.248	0.56	−0.0212 (0.103)	0.84
**Non-targeted Conditions**^**e**^								
All age groups	−0.0034 (0.002)	0.11	−0.2706 (0.049)	**<.0001**	0.0026	0.21	0.006 (0.003)	0.06
18–39 years	0.0071 (0.002)	**0.002**	−0.1247 (0.051)	**0.02**	−0.0031	0.15	−0.0102 (0.003)	**0.002**
40–64 years	0.0087 (0.006)	0.17	−0.1116 (0.107)	0.30	0.0124	**0.05**	0.0037 (0.01)	0.71
≥ 65 years	−0.0216 (0.003)	**<.0001**	−0.4268 (0.071)	**<.0001**	−0.0016	0.59	0.02 (0.004)	**<.0001**

*Abbrev*. *HRRP* Hospital Readmissions Reduction Program, *SE* standard error

^a^ The HRRP announcement phase trend represents the change in rates of readmissions per month before HRRP implementation. This was estimated by calculating the *β*_1_ from the equation

^b^ The level change represents the level change immediately after HRRP implementation. This was estimated by calculating the *β*_2_ from the equation

^c^ The post-HRRP phase trend represents the post-HRRP implementation slope. This was estimated by calculating the sum of *β*_1_ and *β*_3_ from the equation discussed in the methods section

^d^ The difference in trends represents the trend change after HRRP implementation compared to the trend before implementation. This was estimated by calculating the *β*_3_ from the equation

^e^ The interrupted time series analysis was estimated by using the same HRRP implementation time and age restrictions as the acute myocardial infarction, heart failure and pneumoniia cohorts

**Table 4:** DD Estimate of Change in 30-day Readmission Rates Prior to and After Implementation of the Hospital Readmission Reduction Program Between Targeted and Non-targeted Conditions Stratified by Age Groups Using Interrupted Time Series Analysis

	HRRP Announcement Phase Trend^**a**^ (SE)	***P***	Level Change^**b**^ (SE)	***P***	Post-HRRP Phase Trend^**c**^	***P***	Difference in Trends^**d**^ (SE)	***P***
**Acute Myocardial Infarction**								
All age groups	−0.0638 (0.008)	**<.0001**	0.0042 (0.201)	0.98	−0.0373	**<.0001**	0.0265 (0.01)	**0.01**
18–39 years	−0.0228 (0.025)	0.37	−0.0998 (0.635)	0.88	−0.0318	0.19	−0.009 (0.034)	0.8
40–64 years	−0.0491 (0.006)	**<.0001**	−0.0352 (0.158)	0.82	−0.0332	**<.0001**	0.0159 (0.008)	**0.05**
≥ 65 years	−0.0676 (0.014)	**<.0001**	0.1147 (0.338)	0.74	−0.0454	**0.001**	0.0222 (0.021)	0.29
**Heart Failure**								
All age groups	−0.0248 (0.008)	**0.003**	−0.6847 (0.203)	**0.001**	−0.0997	**0.02**	0.0068 (0.011)	0.54
18–39 years	0.0087 (0.037)	0.81	−1.2183 (0.953)	0.21	0.0243	0.49	0.0156 (0.048)	0.74
40–64 years	−0.0353 (0.012)	**0.003**	−0.3339 (0.298)	0.27	−0.0216	**0.05**	0.0137 (0.015)	0.36
≥ 65 years	−0.0093 (0.009)	0.3	−0.6902 (0.224)	**0.003**	−0.019	**0.03**	−0.0097 (0.012)	0.43
**Pneumonia**								
All age groups	−0.0152 (0.006)	**0.02**	−0.2795 (0.141)	**0.05**	−0.0222	**< 0.001**	−0.007 (0.009)	0.43
18–39 years	−0.0061 (0.014)	0.67	0.2977 (0.371)	0.43	−0.004	0.77	0.0021 (0.019)	0.91
40–64 years	0.001 (0.011)	0.92	−0.4609 (0.273)	0.96	−0.0044	0.66	−0.0054 (0.014)	0.69
≥ 65 years	−0.0128 (0.006)	**0.05**	−0.051 (0.164)	0.76	−0.033	**<.0001**	−0.0202 (0.008)	**0.002**
**Chronic Obstructive Pulmonary Disease**^**e**^								
All age groups	−0.0168 (0.008)	**0.04**	0.2957 (0.497)	0.55	−0.028	0.71	−0.0112 (0.076)	0.88
40–64 years	−0.0136 (0.01)	0.19	0.1706 (0.613)	0.78	0.0247	0.8	0.0383 (0.094)	0.68
≥ 65 years	−0.0182 (0.007)	**0.008**	0.4385 (0.54)	0.42	−0.0723	0.37	−0.0541 (0.081)	0.51

*Abbrev*. *DD* difference-in-difference, *HRRP* Hospital Readmissions Reduction Program, *SE* standard error

^a^ The HRRP announcement phase trend represents the change in rates of readmissions per month before HRRP implementation. This was estimated by calculating the *β*_1_ from the equation discussed in the methods section

^b^ The level change represents the level change immediately after HRRP implementation. This was estimated by calculating the *β*_2_ from the equation discussed in the methods section

^c^ The post-HRRP phase trend represents the post-HRRP implementation slope. This was estimated by calculating the sum of *β*_1_ and *β*_3_ from the equation discussed in the methods section

^d^ The difference in trends represents the trend change after HRRP implementation compared to the trend before implementation. This was estimated by calculating the *β*_3_ from the equation discussed in the methods section

^e^ DD analysis was estimated by using the same HRRP implementation time and age restrictions as the Chronic Obstructive Pulmonary Disease cohort

##### Younger population (18–64 years)

Similar patterns of readmission rates decline were found in those aged 40–64 years (decreased from 12.19 to 10.7%) and 18–39 years (decreased from 10.35 to 8.72%) (Table 2**,** Fig. 1**a**). A significant decrease in readmission trends were seen in those aged 40–64 years in the HRRP announcement phase (slope: − 0.0385, *p* < 0.0001), right after the HRRP implementation (level change: − 0.2882, *p* = 0.04), and post-HRRP (slope: − 0.0159, p < 0.0001) phase (Table 3). In DD models, readmission rates decreased significantly in the HRRP announcement (slope: − 0.0491, *p* < 0.0001) and post-HRRP (slope: − 0.0332, *p* < 0.0001) phases (Table 4**,** Fig. 2**a**). Among those aged 18–39 years, there were no significant changes in baseline readmission trends observed in both ITS (HRRP announcement: *p* = 0.54; post-HRRP: *p* = 0.17) and DD models (HRRP announcement: *p* = 0.37; post-HRRP: *p* = 0.19) (Tables 3 and 4).

#### Heart failure

##### Older population (≥ 65 years)

Thirty-day readmission rates in those aged ≥65 years for HF decreased from 24.83 to 22.53% from 2010 to 2015 (Table 2**,** Fig. 1**b**). Readmission rates declined significantly in the HRRP announcement phase (slope: − 0.0275, *p* = 0.008) (Table 3). Directly after the HRRP implementation, there was a significant decrease in readmission rates (level change: − 1.2214, p < 0.0001). In the DD models, the readmission trend decline did not reach statistical significance (slope: − 0.0093, p = 0.3); however, the slope was more negative in the post-HRRP phase (slope: − 0.019, *p* = 0.03) [Table 4**,** Fig. 2**b**]. In addition, a significant level change was observed (level change: − 0.6902, *p* = 0.003).

##### Younger population (18–64 years)

Baseline readmission trends decline were seen in those aged 40–64 years (decreased from 25.89 to 24.64%) (Table 2**,** Fig. 1**b**). For those aged 18–39 years, the trends decreased from 25.79% in 2010 to 24.19% in 2012, going up to 26.30% in 2013, and then decreased to 24.10%. A significant level change was observed in those aged 40–64 years (level change: − 0.6897, *p* = 0.02), with no significant changes in readmission trends in the HRRP announcement (*p* = 0.07) and post-HRRP phases (*p* = 0.82) (Table 3). In the DD models, readmission rates decreased significantly in the HRRP announcement (slope: − 0.0353, *p* = 0.003), and post-HRRP phases (− 0.0216, *p* = 0.05) but no significant level change (*p* = 0.27) was observed (Table 4**,** Fig. 2**b**).

#### Pneumonia

##### Older population (≥ 65 years)

Among those aged ≥65 years hospitalized with pneumonia, the overall 30-day readmission rates decreased from 19.32% in 2010 to 16.76% in 2015 (Table 2**,** Fig. 1**c**). Readmission rates decreased significantly in the HRRP announcement phase (slope: − 0.0339, *p* < 0.0001), immediately after HRRP implementation (level change: − 0.5158, *p* = 0.004), and in the post-HRRP phase (slope: − 0.0328, *p* < 0.0001) (Table 3). In the DD models, readmission rates among those aged ≥65 years declined significantly in the HRRP announcement phase (slope: − 0.0128, *p* = 0.05) and declined further in the post-HRRP phase (slope: − 0.033, *p* < 0.0001) (Table 4**,** Fig. 2**c**).

##### Younger population (18–64 years)

In younger age groups, the baseline readmission trends in those aged 40–64 years increased from 16.96% in 2010 to 17.14% in 2012 and decreased to 16.67% in 2015 (Table 2**,** Fig. 1**c**). Trends in those aged 18–39 years increased from 12.71% in 2010 to 13.05% in 2013 and decreased to 12.85% in 2015. Among those aged 40–64 years, a significantly decrease (level change: − 0.6752, *p* = 0.006) was found after HRRP implementation in the ITS models but did not reached statistical significance (*p* = 0.96) in the DD models (Tables 3 and 4).

#### Acute exacerbation of chronic obstructive pulmonary disease

##### Older population (≥ 65 years)

Readmission rates in those aged ≥65 years for AECOPD decreased from 22.2 to 20.48% between 2010 and 2015 (Table 2**,** Fig. 1**d**). A significant decrease in readmission trends were seen in those aged ≥65 years in the HRRP announcement period (slope: − 0.0360, *p* = 0.005; Table 3). No further significant changes in readmission rates were seen within age groups during the study period. From the DD models, a significant decrease in trends were observed in the HRRP announcement period among those aged ≥65 years (slope: − 0.0182, *p* = 0.008) (Table 4 and Fig. 2**d**).

##### Younger population (18–64 years)

Baseline readmission rates in those aged 40–64 years slightly increased from 21.24% in 2010 to 21.42% in 2015 (Table 2**,** Fig. 1**d**). There were no statistical significance changes observed over the study period from our ITS and DD models in those aged 40–64 years. (Tables 3 and 4).

### Readmission trends for non-targeted conditions prior and after HRRP implementation

Thirty-day readmission rates for non-targeted conditions remained stable through the study time period (Fig. 1**e**, Table 2). Readmission trends in those aged ≥65 years decreased in the HRRP announcement phase (slope: − 0.0216, *p* < 0.0001), and a significant decrease was found immediately after the HRRP implementation (level change: − 0.4268, *p* < 0.0001) (Table 3). For those aged 40–64 years, a significant increase in trend (slope: 0.0124, *p* = 0.05) was seen in the post-HRRP phase. Among those aged 18–39 years, a significant increase in readmission rates were found in the HRRP announcement phase (slope: 0.0071, *p* = 0.002).

### Index hospitalization and readmission costs among the targeted conditions

#### Older population (≥ 65 years)

**Fig. 3:**
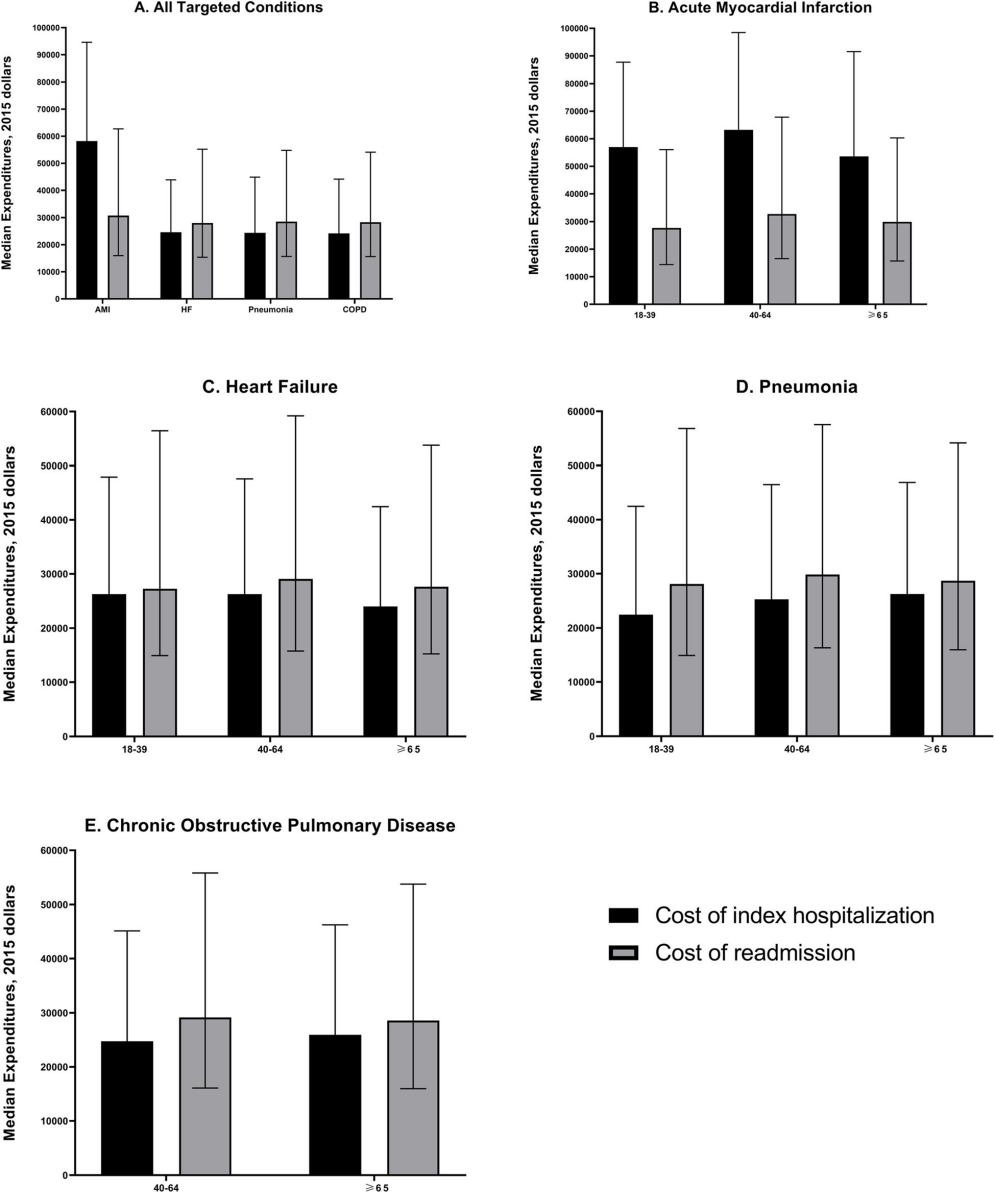
Median Costs of Index Hospitalization and Readmissions Stratified by Targeted Condition and Age Groups. Error bars represent the upper limit of the interquartile rang

Median healthcare expenditures in those aged ≥65 years for readmissions were higher than index hospitalizations for HF, pneumonia, and AECOPD (Fig. 3). For AMI, healthcare expenditures were higher during the index hospitalization. With total expenditures as the outcome, adjusted models showed a significantly higher readmission cost in those readmitted within 30 days (as compared to > 30 days) for AMI ($15,981.08, 95% CI [$13,712.21–$18,444.93], *p* < 0.0001), HF ($16,461.36, 95% CI [$15,275.93–$17,711.23], *p* < 0.0001), pneumonia ($12,622.84, 95% CI [$11,880.55–$13,385.01], *p* < 0.0001), and AECOPD ($10,728.7, 95% CI [$10,074.03–$11,410.21], *p* < 0.0001) (Table 5).

**Table 5:** Cost Difference Between Early (≤30 days) and Late (> 30 days) Readmission Events Stratified by Condition and Age

	Unadjusted model ^**a**^		Adjusted model ^a**,** b^	
	Cost difference (95%CI)	***P***	Cost difference (95%CI)	***P***
**Acute Myocardial Infarction**				
All age groups	$4327.92 ($3902.73 – $4762.70)	< 0.0001	$4974.12 ($4388.94 – $5572.89)	< 0.0001
18–39 years	$-1043.86 ($-3611.68 – $1879.17)	0.47	$-1211.50 ($-2944.96 – $1845.86)	0.37
40–64 years	$3364 ($2604.52 – $4148.07)	< 0.0001	$1403.63 ($982.66 – $1874.51)	< 0.0001
≥ 65 years	$5044 ($4522.25 – $5571.34)	< 0.0001	$15,981.08 ($13,712.21 – $18,444.93)	< 0.0001
**Heart Failure**				
All age groups	$6448.47 ($6260.52 – $6753.10)	< 0.0001	$8214.33 ($7805.49 – $8631.4)	< 0.0001
18–39 years	$13,362.79 ($11,110.16 – $15,762.72)	< 0.0001	$20,035.9 ($15,401.37 – $25,548.43)	< 0.0001
40–64 years	$ 7512.03 ($6969 – $8066.45)	< 0.0001	$4623.18 ($4157.55 – $5120.16)	< 0.0001
≥ 65 years	$ 5752.42 ($5481.69 – $6029.36)	< 0.0001	$16,461.36 ($15,275.93 – $17,711.23)	< 0.0001
**Pneumonia**				
All age groups	$8856.43 ($8610.4 – $9109.65)	< 0.0001	$8027.16 ($7726.24 – $8338.59)	< 0.0001
18–39 years	$12,745.66 ($11,419.96 – $14,122.99)	< 0.0001	$12,867.63 ($10,991.28 – $14,937.81)	< 0.0001
40–64 years	$9890.97 ($9373.67 – $10,419.55)	< 0.0001	$6020.75 ($5523.78 – $6553)	< 0.0001
≥ 65 years	$8228.76 ($7944.76 – $8518.59)	< 0.0001	$12,622.84 ($11,880.55 – $13,385.01)	< 0.0001
**Chronic Obstructive Pulmonary Disease**				
All age groups	$8390.38 ($8148.52 – $8633.39)	< 0.0001	$6642.03 ($6391.26 – $6901.31)	< 0.0001
40–64 years	$7754.06 ($7283.05 – $8229.44)	< 0.0001	$5107.02 ($4664.35 – $5562.89)	< 0.0001
≥ 65 years	$7751.64 ($7464.94 – $8039.25)	< 0.0001	$10,728.7 ($10,074.03 – $11,410.21)	< 0.0001

^a^ Standardized to 2015 U.S. dollars

^b^ Adjusted for age, gender, median household income, and Elixhauser comorbidity index scores

#### Younger population (18–64 years)

Among younger age groups median healthcare expenditures for readmissions were higher than index hospitalizations for HF, pneumonia, AECOPD (Fig. 3). The median expenditures of patients aged 40–64 years for readmissions were higher than other age groups across all targeted conditions. For total expenditures, the cost differences among those aged 18–39 years for HF ($20,035.9, 95%CI [$15,401.37–$25,548.43], *p* < 0.0001) and pneumonia ($12,867.63, 95%CI [$10,991.28–$14,937.81], *p* < 0.0001) were larger than other age groups (Table 5).

## Discussion

This study used a nationwide representative dataset from 2010 to 2015 to provide estimates of hospital readmissions for the major four HRRP conditions prior to and after policy implementation. Overall, 30-day readmission rates in those aged ≥65 years for AMI and pneumonia decreased before and after policy implementation based on ITS and DD models. Similar trend patterns were found in those aged 40–64 years for AMI in both models, and for HF in the DD models only. After adjustment for covariates, healthcare expenditures in those aged ≥65 years and 40–64 years were significantly higher for those readmitted within 30 days for all targeted indications. The cost differences of healthcare expenditures were largest in those aged 18–39 years for HF and pneumonia.

Previous studies have examined the patient characteristics of readmission patterns among younger adults in the original 3 targeted conditions (AMI, HF, and pneumonia) but limited to single year or selected state [6, 7]. Studies also reported readmission burden in younger adults for those 3 conditions, approximately 1 in 12 patients with AMI, 1 in 5 patients with HF, and 1 in 7 patients with pneumonia aged < 65 years were readmitted within 30-days of discharge [7, 25]. Our results extend previous articles and also support Angraal et al. work by confirming the decreased readmission trends before and after the HRRP implementation among younger and older populations [26]. We further stratified the younger populations into 2 age groups (18–39 years and 40–64 years) for three targeted conditions and the rates of readmission decline were observed in those aged 40–64 years for AMI in ITS and DD models, and for HF in DD models (Tables 3 and 4). Myers et al. examined the association between the AECOPD and HRRP and showed a modest decrease in all-cause readmission prior to AECOPD being included as a targeted diagnosis, which is consistent with our findings [27]. Furthermore, our results include larger sample size than previous studies and perform the longitudinal analysis in younger adults (40–64 years) and older adults for AECOPD separately and find similar patterns of decreased readmission rates in both populations in the HRRP announcement phase [27, 28]. We believe it is possibly that reducing readmissions was already a nationwide concern far before the PPACA and hospitals already responded to reduce readmission rates before policy implementation.

There is currently limited literature that evaluates the cost burden of hospitalizations and readmissions for AMI, HF, pneumonia, and AECOPD. A study by Jain et al. used the NRD to evaluate pneumonia hospitalizations and found mean cost per readmission was highest for the 40–64 age group ($15,976) [29]. A separate study evaluated COPD using the Florida State Inpatient Database and found mean total charges for 30-day COPD related readmissions increased from 2009 ($36,714) to 2014 ($40,611; *p* = 0.011) [30]. Finally Khera et al. evaluated AMI using the NRD and found cumulative costs of 30 day readmissions was $1.1 billion, of which $365 million was among those < 65 years of age [25]. Our findings extend this work to the four targeted therapeutic conditions including older and younger age groups. We show that the median costs of index hospitalizations among younger age groups are slightly higher or close to the costs in the older populations across the four targeted conditions. This supports that the cost burden to the healthcare system among younger populations is substantial and should be addressed in future research. Based on our adjusted cost difference models, we found that healthcare expenses differences are seen within therapeutic condition as well as specific age groups. These factors should be accounted for in future policy with necessary changes made periodically based on the outcomes in each condition and age group in order to decrease healthcare costs.

The HRRP financially incentivizes hospitals to reduce excess readmissions among fee-for-service Medicare patients aged ≥65 years [2, 3]. Previous studies have shown a decrease in 30-day hospital readmissions in Medicare patients for the initial targeted conditions (e.g., AMI, HF, and pneumonia) under the HRRP. Our baseline trends of 30-day all-cause readmission rates are consistent with the existing literature for these initial targeted conditions among older populations. Whether the decrease in readmission rates is due to the HRRP policy or alternative explanations is controversial. McWilliams et al. suggested a concurrent decline in admission rates during this time period, which may explain much of the reduction in readmission rates for targeted and non-targeted conditions by the HRRP [31]. Another proposed explanation is that the majority of the decrease in readmission was generated by increased patient risk scores, rather than by actual lower readmission rates [32, 33]. The Centers for Medicare and Medicaid Services changed the electronic standards that hospitals used to submit Medicare claims starting in 2011. This increased the number of secondary diagnoses reporting from 10-25 diagnosis codes, which allowed for the coding of additional comorbid conditions. After accounting for this change, this would reduce the decline in risk-adjusted readmission rates for targeted conditions by 48%. In addition, we found that readmission rates declined in select age groups outside of the targeted ≥65 years Medicare group. One potential reason for declining readmission rates for those < 65 years may be due to a spillover effect of the policy beyond the Medicare population, which has been previously described in non-targeted Medicare conditions, Medicaid and private insurance populations [8, 26, 34, 35]. Our findings support the decline in readmission rates from 2010 to 2015 for targeted conditions among older adults and in some cases younger adults. The extent to which these declines are due to the HRRP policy or attributed to alternative explanations is still unclear.

The HRRP policy expanded in October 2014 to include AECOPD; however, at the time, there was a lack of published evidence that pointed to effective hospital-based programs to reduce COPD-related readmissions [36]. Given the financial penalties instituted by the CMS for 30-day readmissions, it is probable that hospitals across the U.S. were implementing quality improvement programs in these targeted areas. Interestingly, several studies have shown limited success at reducing readmissions after a hospitalization for COPD. Jennings et al. performed a single-center, randomized clinical trial evaluating a pre-discharge bundle for COPD exacerbations using a low resource approach and found no differences in readmission rates [37]. A significant limitation was that a tool designed to identify COPD readmission risk factors was ineffective, as it did not include specific interventions or patient follow-up; interventions for identified risk factors were left to the judgement of the primary team. Bhatt et al. performed a single-center, pre-post intervention study evaluating a comprehensive COPD multidisciplinary intervention focusing on inpatient, transitional, and outpatient care [38]. The intervention did not reduce 30-day readmission rates or overall costs. Finally, Aboumatar et al. performed a single-center, randomized clinical trial, initiating a three-month program that combined transition support with long-term disease self-management support for patients admitted for AECOPD [39]. The program resulted in significantly more COPD-related hospitalizations and emergency department visits without improvements in quality of life. A sustained decrease in COPD-related readmissions may be interrupted without specific evidence-based interventions showing success in reducing these readmissions. There is a need for continued surveillance as additional data become available and the impact of the HRRP on AECOPD readmissions can be further elucidated.

The penalties associated with the HRRP are quite high, as hospitals could lose up to 3% of their total fee-for-service Medicare payments due to poor performance on the readmission metric [2]. Readmission-specific incentives and initiatives may have contributed to the decrease in readmissions rates for the targeted conditions. As programs were announced to curb readmission rates, the added interventions may have influenced patient care within Medicare or beyond Medicare patients through increased awareness of changes in the discharge process. For example, Ody et.al reported that changing the CMS Medicare electronic transaction standards allows documenting more diagnosis codes per claim between 2010 and 2012, which may have led to changes on nationwide readmissions and current coding practice in hospitals [32]. Even with the pronounced decrease in readmissions since policy implementation, 83% of hospitals were penalized under this program for fiscal year 2020 [3]. In addition, evidence is emerging that post-discharge healthcare utilization is increasing (e.g., observation units and emergency department visits) with the subsequent decrease in inpatient readmissions [40]. These increases could be attributable to hospitals treating patients who return within 30 days of discharge in these units, as these are not evaluated under the HRRP policy. Further research is necessary evaluating total healthcare utilization following hospital discharge including emergency department visits, observation units, and hospital readmissions in order to better understand the overall impact of the HRRP on the U.S. healthcare system.

The findings of our study should be interpreted in the context of important limitations. First, we relied on CMS algorithms using ICD-9-CM codes to classify hospitalizations for our targeted cohorts. The selections for the ICD-9-CM codes based algorithms may have led to an underestimation of the number of hospitalizations for the targeted conditions [41]. Since this methodology is used by the CMS to identify hospital admissions, we felt it was prudent to apply it to the entire study to provide national readmission estimates across age groups. Due to a limitation in the NRD, we excluded patients who were residents of different states. Persons are identified and tracked in the NRD by state-specific linkage numbers; therefore, a person readmitted between two different states cannot be tracked between states. In addition, we did not include data after October 1, 2015, since the United States newly implemented the ICD-10-CM coding system. It is uncertain whether the transition from ICD-9-CM to ICD-10-CM affects the coding of our targeted conditions. In addition, the first year of available NRD data is 2010, therefore we could not account for readmission rate changes in the pre-2010 period. Previous research has shown a readmission trend change in 2010 as a consequence of the passage of the PPACA [3]. Due to the nature of the NRD, the hospitalizations can only be linked within a year. Thus, the follow up window of late readmission for index hospitalizations occurred later in the year may not be consistent which may lead to under/overestimate the results of the cost difference between early and late readmissions. In 2016, the twenty-first Century Cures Act introduced a new payment method for the HRRP program starting fiscal year 2019 (October 1, 2018 to September 30, 2019) [42–44]. Our study did not reflect this new payment method, however future longitudinal research is needed to evaluate the performance of this new policy for all targeted conditions and age groups.

## Conclusions

Readmissions in those aged ≥65 years for targeted conditions including AMI and pneumonia decreased in the U.S. prior to and after implementation of the HRRP policy. A decrease in hospital readmissions was seen in younger age groups (aged 40–64 years) for AMI and HF not originally targeted by the program. Healthcare expenditures in those aged ≥65 years and 40–64 years for early readmissions were significantly higher for all targeted conditions. Further research is needed into whether a similar downward trend is occurring beyond hospital admissions including additional markers of healthcare utilization such as observation unit and emergency department visits.

## Supplementary Information

**Additional file 1: Table S1.** ICD-9-CM Codes Used to Define Acute Myocardial Infarction Cohort. **Table S2.** ICD-9-CM Codes Used to Define Heart Failure Cohort. **Table S3.** ICD-9-CM Codes Used to Define Pneumonia Cohort. **Table S4.** ICD-9-CM Codes Used to Define Chronic Obstructive Pulmonary Disease. Cohort. **Table S5.** Baseline Patient Characteristics of Index Admissions by Age Group, Insurance Type, and Targeted Condition. **Table S1-S4** include the ICD-9-CM codes for each cohort base on those published by the Centers for Medicare and Medicaid Services for the HRRP for assessment of all cause readmissions. **Table S5 **includes further breakdowns for patient characteristics of index admissions by age, insurance, and condition.

## Data Availability

All relevant data is available from the Nationwide Readmissions Database through the AHRQ Healthcare Cost and Utilization Project. The data are publicly available and could be purchased online through the AHRQ.

## Abbreviations

HRRP: Hospital readmissions reduction program
PPACA: Patient protection and affordable care act
AMI: Acute myocardial infarction
HF: Heart failure
AECOPD: Acute exacerbations of chronic obstructive pulmonary disease
FY: Fiscal year
NRD: Nationwide Readmission Database
AHRQ: Agency for Healthcare Research and Quality
HCUP: Healthcare Cost and Utilization Project
ICD-9-CM: *International Classification of Diseases, Ninth Revision, Clinical Modification*
CMS: Centers for Medicare and Medicaid Services
DD: Difference-in-difference
