# Supplementary material for: Readmissions and costs among younger and older adults for targeted conditions during the enactment of the hospital readmission reduction program
Source: BMC Health Serv Res. 2021 Apr 26;21:386. doi: 10.1186/s12913-021-06399-z (PMC8077835; doi:10.1186/s12913-021-06399-z)
Supplement: Supplementary file 1 — Additional file 1: Table S1. ICD-9-CM Codes Used to Define Acute Myocardial Infarction Cohort. Table S2. ICD-9-CM Codes Used to Define Heart Failure Cohort. Table S3. ICD-9-CM Codes Used to Define Pneumonia Cohort. Table S4. ICD-9-CM Codes Used to Define Chronic Obstructive Pulmonary Disease. Cohort. Table S5. Baseline Patient Characteristics of Index Admissions by Age Group, Insurance Type, and Targeted Condition. Table S1-S4 include the ICD-9-CM codes for each cohort base on those published by the Centers for Medicare and Medicaid Services for the HRRP for assessment of all cause readmissions. Table S5 includes further breakdowns for patient characteristics of index admissions by age, insurance, and condition. [file 12913_2021_6399_MOESM1_ESM.docx]

| **S1 Table. ICD-9-CM Codes Used to Define Acute Myocardial Infarction Cohort** | |
| --- | --- |
| **ICD-9-CM Diagnosis Codes** | **Description** |
| 410.00 | Acute myocardial infarction of anterolateral wall, episode of care unspecified |
| 410.01 | Acute myocardial infarction of anterolateral wall, initial episode of care |
| 410.10 | Acute myocardial infarction of other anterior wall, episode of care unspecified |
| 410.11 | Acute myocardial infarction of other anterior wall, initial episode of care |
| 410.20 | Acute myocardial infarction of inferolateral wall, episode of care unspecified |
| 410.21 | Acute myocardial infarction of inferolateral wall, initial episode of care |
| 410.30 | Acute myocardial infarction of inferoposterior wall, episode of care unspecified |
| 410.31 | Acute myocardial infarction of inferoposterior wall, initial episode of care |
| 410.40 | Acute myocardial infarction of other inferior wall, episode of care unspecified |
| 410.41 | Acute myocardial infarction of other inferior wall, initial episode of care |
| 410.50 | Acute myocardial infarction of other lateral wall, episode of care unspecified |
| 410.51 | Acute myocardial infarction of other lateral wall, initial episode of care |
| 410.60 | True posterior wall, episode of care unspecified |
| 410.61 | True posterior wall, initial episode of care |
| 410.70 | Subendocardial infarction, episode of care unspecified |
| 410.71 | Subendocardial infarction, initial episode of care |
| 410.80 | Acute myocardial infarction of other specified site, episode of care unspecified |
| 410.81 | Acute myocardial infarction of other specified site, initial episode of care |
| 410.90 | Acute myocardial infarction of unspecified site, episode of care unspecified |
| 410.91 | Acute myocardial infarction of unspecified site, initial episode of care |

| **S2 Table. ICD-9-CM Codes Used to Define Heart Failure Cohort** | |
| --- | --- |
| **ICD-9-CM Diagnosis Codes** | **Description** |
| 402.01 | Malignant hypertensive heart disease with congestive heart failure |
| 402.11 | Benign hypertensive heart disease with congestive heart failure |
| 402.91 | Hypertensive heart disease with congestive heart failure |
| 404.01 | Malignant hypertensive heart and renal disease with congestive heart failure |
| 404.03 | Malignant hypertensive heart and renal disease with congestive heart failure & renal failure |
| 404.11 | Benign hypertensive heart and renal disease with congestive heart failure |
| 404.13 | Benign hypertensive heart and renal disease with congestive heart failure & renal failure |
| 404.91 | Unspecified hypertensive heart and renal disease with congestive heart failure |
| 404.93 | Hypertension and non-specified heart and renal disease with congestive heart failure & renal failure |
| 428.xx | Heart failure codes |

| **S3 Table. ICD-9-CM Codes Used to Define Pneumonia Cohort** | |
| --- | --- |
| **ICD-9-CM Diagnosis Codes** | **Description** |
| 480.0 | Pneumonia due to adenovirus |
| 480.1 | Pneumonia due to respiratory syncytial virus |
| 480.2 | Pneumonia due to parainfluenza virus |
| 480.3 | Pneumonia due to SARS-associated coronavirus |
| 480.8 | Viral pneumonia: pneumonia due to other virus not elsewhere classified |
| 480.9 | Viral pneumonia unspecified |
| 481 | Pneumococcal pneumonia [streptococcus pneumoniae pneumonia] |
| 482.0 | Pneumonia due to klebsiella pneumoniae |
| 482.1 | Pneumonia due to pseudomonas |
| 482.2 | Pneumonia due to hemophilus influenzae |
| 482.30 | Pneumonia due to streptococcus unspecified |
| 482.31 | Pneumonia due to streptococcus group a |
| 482.32 | Pneumonia due to streptococcus group b |
| 482.39 | Pneumonia due to other streptococcus |
| 482.40 | Pneumonia due to staphylococcus unspecified |
| 482.41 | Pneumonia due to staphylococcus aureus |
| 482.49 | Other staphylococcus pneumonia |
| 482.81 | Pneumonia due to anaerobes |
| 482.82 | Pneumonia due to escherichia coli |
| 482.83 | Pneumonia due to other gram-negative bacteria |
| 482.84 | Pneumonia due to legionnaires' disease |
| 482.89 | Pneumonia due to other specified bacteria |
| 482.9 | Bacterial pneumonia unspecified |
| 483.0 | Pneumonia due to mycoplasma pneumoniae |
| 483.1 | Pneumonia due to chlamydia |
| 483.8 | Pneumonia due to other specified organism |
| 485 | Bronchopneumonia organism unspecified |
| 486 | Pneumonia organism unspecified |
| 487.0 | Influenza with pneumonia |

| **S4 Table. ICD-9-CM Codes Used to Define Chronic Obstructive Pulmonary Disease Cohort** | |
| --- | --- |
| **ICD-9-CM Diagnosis Codes** | **Description** |
| 491.21 | Obstructive chronic bronchitis with (acute) exacerbation |
| 491.22 | Obstructive chronic bronchitis; with acute bronchitis |
| 491.8 | Other chronic bronchitis. Chronic: tracheitis, tracheobronchitis. |
| 491.9 | Unspecified chronic bronchitis |
| 492.8 | Other emphysema |
| 493.20 | Chronic obstructive asthma, unspecified |
| 493.21 | Chronic obstructive asthma with status asthmaticus |
| 493.22 | Chronic obstructive asthma with (acute) exacerbation |
| 496 | Chronic airway obstruction, not elsewhere classified |
| 518.81* | Other diseases of lung; acute respiratory failure; respiratory failure NOS |
| 518.82* | Other diseases of lung; acute respiratory failure; other pulmonary insufficiency, acute respiratory distress |
| 518.84* | Other diseases of lung; acute respiratory failure; acute and chronic respiratory failure |
| 799.1* | Other ill-defined and unknown causes of morbidity and mortality; respiratory arrest, cardiorespiratory failure |
| *Principal diagnosis when combined with a secondary diagnosis of AECOPD (491.21, 491.22, 493.21, or 493.22) | |

| **S5 Table. Baseline Patient Characteristics of Index Admissions by Age Group, Insurance Type, and Targeted Condition** | | | | | | | | | | | |
| --- | --- | --- | --- | --- | --- | --- | --- | --- | --- | --- | --- |
| **Characteristics** | **Acute Myocardial Infarction**  **No. (%)** | | | **Heart Failure**  **No. (%)** | | | **Pneumonia**  **No. (%)** | | | **COPD**  **No. (%)** | |
| **Age Group** | **18-39** | **40-64** | **≥65** | **18-39** | **40-64** | **≥65** | **18-39** | **40-64** | **≥65** | **40-64** | **≥65** |
| **Total N** | 55,888 | 1,006,237 | 1,316,881 | 104,606 | 1,152,003 | 3,225,634 | 379,358 | 1,616,175 | 3,805,654 | 1,638,215 | 2,541,953 |
| **Medicare** | 4,142  (7.4) | 163,654 (16.3) | 1,190,260 (90.4) | 21,454  (20.5) | 407,449  (35.4) | 2,987,317  (92.6) | 58,198  (15.4) | 565,928  (35.0) | 3,524,745  (92.6) | 626,622  (38.0) | 2,335,057  (91.8) |
| **Medicaid** | 12,253 (21.9) | 137,340 (13.6) | 15,959  (1.2) | 40,441  (38.7) | 306,863  (26.6) | 45,520  (1.4) | 123,737  (32.7) | 340,007  (21.0) | 50,792  (1.3) | 478,343  (29.0) | 37,456  (1.5) |
| **Private** | 23,980 (42.9) | 505,376 (50.2) | 89,224  (6.8) | 20,333  (19.4) | 276,413  (24.0) | 148,201  (4.6) | 122,266  (32.3) | 514,081  (31.8) | 179,963  (4.7) | 332,265  (20.2) | 129,640  (5.1) |
| **Self-pay** | 11,629 (20.8) | 125,559 (12.5) | 3,848  (0.3) | 16,408  (15.7) | 100,668  (8.7) | 8,246  (0.3) | 53,364  (14.1) | 114,349  (7.1) | 7,882  (0.2) | 124,185 (7.5) | 5,025  (0.2) |
| **Other** | 3,884 (7.0) | 74,308 (7.4) | 17,590  (1.3) | 5,970  (5.7) | 60,610  (5.3) | 36,350  (1.1) | 20,793 (5.5) | 81,810  (5.1) | 42,272  (1.2) | 86,500  (5.3) | 34,775  (1.4) |
| Abbrev. COPD, chronic obstructive pulmonary disease; | | | | | | | | | | | |
